# Altered of apoptotic markers of both extrinsic and intrinsic pathways induced by hepatitis C virus infection in peripheral blood mononuclear cells

**DOI:** 10.1186/1743-422X-9-314

**Published:** 2012-12-20

**Authors:** Guilherme Albertoni, Carine Prisco Arnoni, Flávia Roche Moreira Latini, Sheila Siqueira Andrade, Patrícia Regina Barboza Araújo, Patrícia Bortman Rozenchan, Maria Cássia Mendes-Correa, Olavo Henrique Munhoz Leite, Nestor Schor, Manoel João Castelo Batista Girão, José Augusto Barreto

**Affiliations:** 1 Colsan - Associação Beneficente de Coleta de Sangue, São Paulo, SP, Brazil; 2 Department of Gynecology, Federal University of São Paulo (UNIFESP), São Paulo, SP, Brazil; 3 URDIP - Unidade de Referência para Doenças Infecciosas Preveníveis, São Paulo, SP, Brazil; 4 Department of Nephrology, Federal University of São Paulo (UNIFESP), São Paulo, SP, Brazil

**Keywords:** HCV infection, Apoptosis, Caspase 3, Caspase 8, Caspase 9

## Abstract

**Background:**

Chronic hepatitis C (CHC) has emerged as a leading cause of cirrhosis in the U.S. and across the world. To understand the role of apoptotic pathways in hepatitis C virus (HCV) infection, we studied the mRNA and protein expression patterns of apoptosis-related genes in peripheral blood mononuclear cells (PBMC) obtained from patients with HCV infection.

**Methods:**

The present study included 50 subjects which plasma samples were positive for HCV, but negative for human immunodeficiency virus (HIV) or hepatitis B virus (HBV). These cases were divided into four groups according to METAVIR, a score-based analysis which helps to interpret a liver biopsy according to the degree of inflammation and fibrosis. mRNA expression of the studied genes were analyzed by reverse transcription of quantitative polymerase chain reaction (RT-qPCR) and protein levels, analyzed by ELISA, was also conducted. HCV genotyping was also determined.

**Results:**

HCV infection increased mRNA expression and protein synthesis of caspase 8 in **group 1** by 3 fold and 4 fold, respectively (p < 0.05). In **group 4** HCV infection increased mRNA expression and protein synthesis of caspase 9 by 2 fold and 1,5 fold, respectively (p < 0.05). Also, caspase 3 mRNA expression and protein synthesis had level augumented by HCV infection in **group 1** by 4 fold and 5 fold, respectively, and in **group 4** by 6 fold and 7 fold, respectively (p < 0.05).

**Conclusions:**

HCV induces alteration at both genomic and protein levels of apoptosis markers involved with extrinsic and intrinsic pathways.

## Background

Hepatitis C virus (HCV) is a major worldwide causative pathogen of chronic hepatitis (CHC), cirrhosis, and hepatocellular carcinoma 1. Although the exact mechanisms governing the elements of HCV pathogenesis, such as viral persistence, hepatocyte injury, and hepatocarcinogenesis, are not fully understood, an accumulating body of evidence suggests that apoptosis of hepatocytes and peripheral blood mononuclear cells (PBMCs) are significantly involved 2,3. Apoptosis plays a pivotal role in the maintenance of cellular homeostasis through removal of aged, damaged, and hyperproliferative cells 4. Resistance to apoptosis induced by various stimuli is one of the most important factors in tumor progression, as well as in resistance to cytotoxic therapies 5. In mammalian cells, apoptosis can be induced via two major pathways. One is the death receptor pathway (extrinsic pathway), which is triggered by binding of FAS ligand (FASLG) to FAS (CD95). This leads to activation of caspase 8 (CASP8), which subsequently activates effector caspases 3, 6, and 7 (CASP3, CASP6, and CASP7) 6,7,8,9. The second apoptosis pathway (intrinsic pathway) is induced by mitochondria in response to DNA damage, oxidative stress, and viral proteins 2. Mitochondrion-dependent apoptosis is mediated by pro-apoptotic genes (*BAX*, *BAD*, *BAK*, and others), whereas proteins like BCL-2 and BCL-XL are anti-apoptotic. These proteins converge at the mitochondrial permeability transition pore, which regulates the release of apoptotic regulatory proteins such as procaspase-9 and cytochrome c (CYCS) 10.

Some studies have indicated that apoptosis of hepatocytes plays a significant role in the pathogenesis of HCV infection 11,12,13,14, which is clinically recognized as liver inflammation and fibrosis. To date, other alternative sites of HCV replication, such as the PBMCs have been recognized 15,16. Therefore, CHC should be considered as a systemic disease rather than a local one 8. Moreover, the HCV ability to directly infect those cells might affect their function and down-modulate apoptotic events, thereby allowing virus chronic replication in target cells. However, the susceptibility of PBMCs to apoptotic process in course of chronic HCV infection has not yet been fully understood 13. Therefore, the aim of this work was to evaluate in PBMCs obtained from chronic HCV patients the genomic and protein expression of 3, 8, 9 capases as well as Fas and, CYCS which are involved with intrinsic and extrinsic apoptotic pathways.

## Results

### Clinical aspects

The present study includes a total of 100 subjects, being 50 of them infected by HCV virus. These patients were divided into four groups, according to the degree of fibrosis and inflammation, scored by METAVIR. In the control group, 25 men and 25 women were selected. Subjects with diabetes mellitus, hypertension, connective tissue disease, other systemic diseases, acute or chronic inflammatory disorders, infections, or hepatocellular carcinoma or other malignancy, as well as alcoholics and smokers, were excluded. All patients signed informed consents before inclusion and the ethical committee has approved the protocol, which was in accordance with the ethical guidelines of 1975 Declaration of Helsinki. Table 1 shows the patients distribution according clinical features.

**Table 1:** Patients characteristics/variables

**Parameter/Variables Mean ± S.D.**	**Group 1 (*n*=*13*)**	**Group 2 (*n*=*13*)**	**Group 3 (*n*=*13*)**	**Group 4 (*n*=*11*)**	**Control Group (*n*=*50*)**
**ALT (mg/dl)**	32.1 ± 4.7*	21.2 ± 2.8	19.9 ± 5.0	101.8 ± 5.0**	17.2 ± 6.1
**AFP (ng/ml)**	39.47 ± 0.2**	13.65 ± 0.1	16.82 ± 0.8**	56.76 ± 0.5**	10.03 ± 0.1
**Viral load (IU/ml)**	66.571 ± 3196	495.358 ± 2821	3.568.588 ± 2207	11.174.667 ±6385	-
**Age (Mean)**	47.8 ± 2.05	44.1 ± 1.58	46.9 ± 1.59	55.0 ± 2.98	45.0 ± 2.4
**Male:female ratio**	1:1	1.8:1	2.3:1	6.0:1	1:1
**Genotype**	1b/1a (73,13%)		3 (26,87%)		

ALT, alanine aminotransferase (ALT normal range = Less than 20 mg/dl (Park *et al*., 2011); AFP, α-fetoprotein (AFP normal range = Less than 20 ng/ml (Sato *et al*., 1993); (*) p< 0.05 vs. CTL; (**) p < 0.0001 vs. CTL.

### Alterations in apoptotic markers in PBMCs

**Figure 1:**
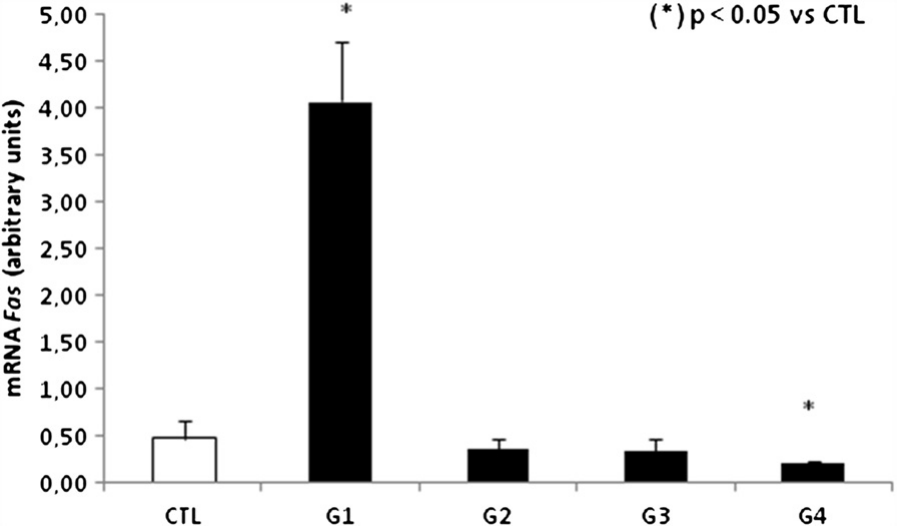
**Analysis of mRNA expression of *FAS *in PBMCs by qPCR.**

**Figure 2:**
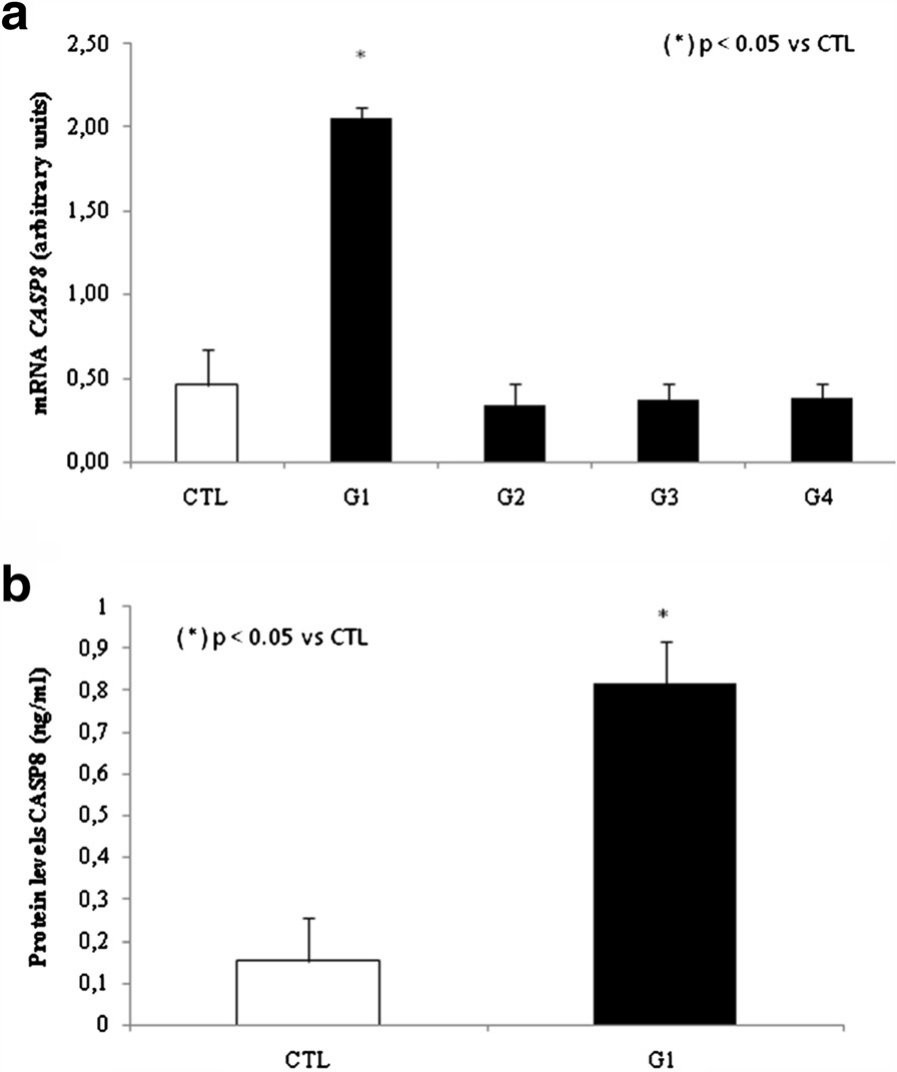
**a. Analysis of mRNA expression *CASP8 *in PBMCs by qPCR. b.** Analysis of protein levels of CASP8 in plasma.

We first analyzed genes involved with the extrinsic apoptosis pathway by measuring the expression levels of *FAS* and *CASP8* in PBMCs. In group 1, we observed a 7.5 fold increase in relative *FAS* expression compared to control group, p < 0.05. However, *FAS* expression was decreased by 5 fold in group 4 compared to control group, p < 0.05 (Figure 1). We did not observe any significant difference in *FAS* expression when comparing groups 2 and 3 with the control group. We then analyzed *CASP8* relative expression and observed a statistically significant increase of 3 fold change for group 1 when compared to control group, p < 0.05 (Figure 2a).

To validate these results, we decided to analyze CASP8 at protein level and observed, by ELISA, that only group 1 has CASP8 increased by 4 fold when compared to control group, p < 0.05 (Figure 2b).

**Figure 3:**
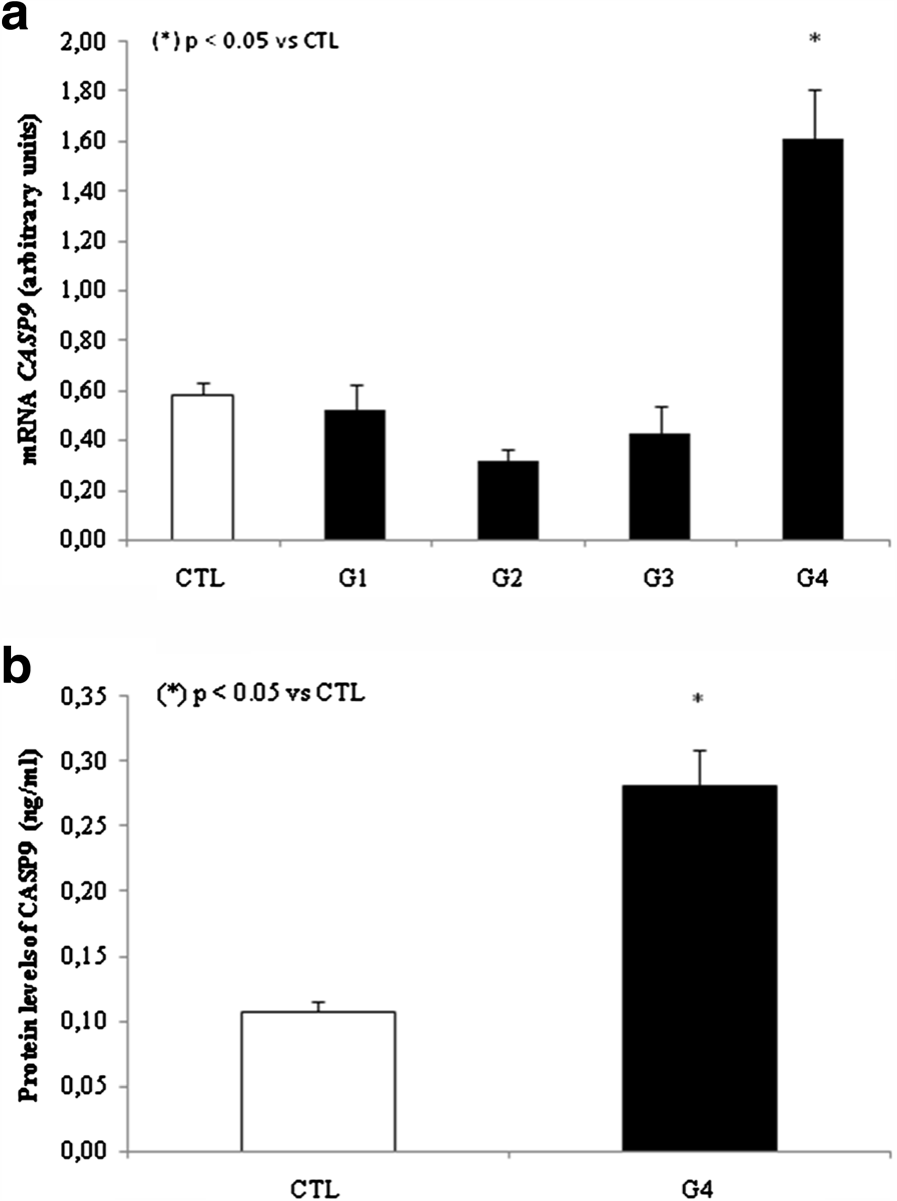
**a. Analysis of mRNA expression *CASP9 *in PBMCs by qPCR. b.** Analysis of protein levels of CASP9 in plasma.

**Figure 4:**
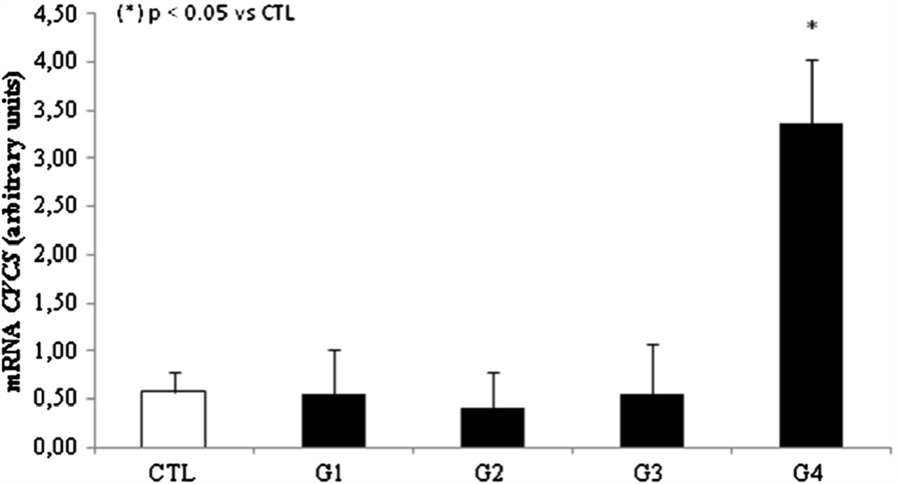
Analysis of mRNA expression CYCS in PBMCs by qPCR.

We next investigated whether intrinsic apoptosis could possibly be involved in HCV infection through analysis of relative expression of *CASP9* and *CYCS* in PBMCs. We observed that both were significantly increased in group 4 when compared to the control group. Specifically, there was an increase of 2 fold in *CASP9* expression and 5 fold change in *CYCS* expression (Figures 3a and 4). No differences were observed for groups 1, 2, or 3 when compared to control group. To confirm our results, we also evaluated CASP9 protein levels in group 4 and the control group, and found that the difference in mRNA expression was reflected in the protein levels. Specifically, we observed a 1.5 fold increase in group 4 over the control group, p < 0.05 (Figure 3b).

**Figure 5:**
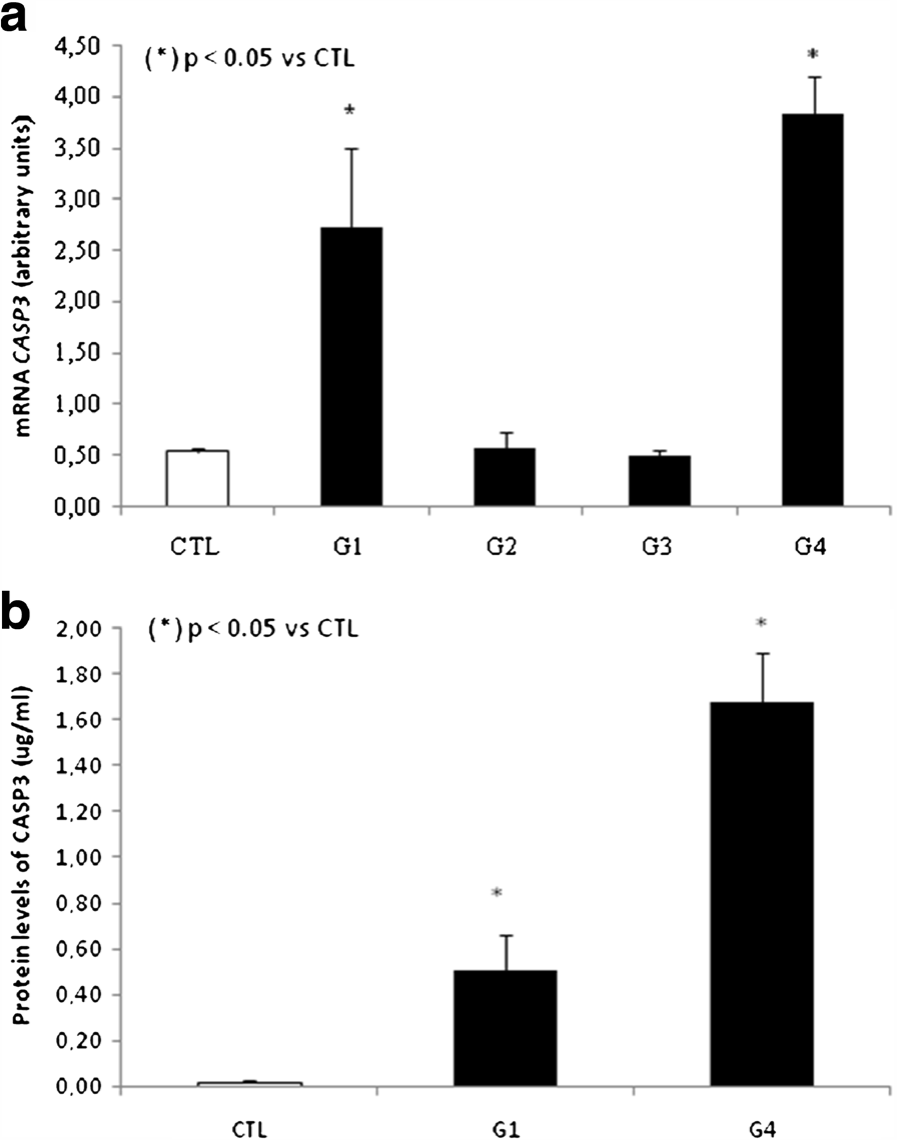
**a. Analysis of mRNA expression *CASP3 *in PBMCs by qPCR. b.** Analysis of protein levels of CASP3 in plasma. Data were expressed as the mean ± standard error of the mean. Experimental and control groups were compared via the Student’s t-test and ANOVA test. The significance level for a null hypothesis was set at 5% (p < 0.05).

Since *CASP3* increased levels has already been associated with activation of both the extrinsic and intrinsic apoptosis pathways 2, we also decided to analyze this caspase at genomic and protein level. mRNA expression showed a 4 fold increase in group 1, p < 0.05 and 6 fold increase for group 4 in *CASP3* expression levels compared to control group (Figure 5a). 

To measure CASP3 protein levels we standardized an “in-house” ELISA because a commercial assay kit for this protein was not available. As expected, we found an augment in groups 1 (5 fold increase, p < 0.05) and 4 (7 fold increase, p < 0.05) when compared to control group. (Figure 5b).

## Discussion

HCV often establishes itself as a persistent infection that causes CHC, liver cirrhosis, and hepatocellular carcinoma, which are significant health problems throughout the world 1. Despite increasing knowledge of the molecular virology of HCV and associated apoptosis markers, the mechanisms of hepatocellular injury in HCV infection are not fully understood. A delicate balance normally exists in cells between antiapoptotic and proapoptotic regulators to ensure the proper survival and turnover of different body cells 17.

In our study, we analyzed the influence of liver inflammation and fibrosis in CHC patients on the gene expression of *FAS* receptor, *CASP3*, *CASP8*, *CASP9*, and *CYCS* in PBMCs. Further, we examined protein levels of CASP3, CASP8, and CASP9 in plasma samples. These factors are actively involved in apoptotic cell death, and could indirectly indicate the susceptibility of cells to apoptotic death. The apoptotic process seems to be the host’s defense mechanism against viral infections, resulting in interruption of viral replication and elimination of infected cells. FASL presented on activated CD8^+^ cytotoxic T cells interacts with overexpressed FAS receptors to trigger apoptosis in infected hepatocytes and PBMCs. The mechanisms for persistence of HCV infection are complex because of divergent virus strategies for immune system evasion. Under physiological conditions, FAS-FASLG interactions protect immunological homeostasis by regulating apoptosis of various immune cells 18.

We observed significantly increased expression of *FAS* receptor in PBMCs in patients from group 1. *FAS* receptor expression may represent a self-limiting mechanism of the immune response 19. HCV genotype-dependent differences in FAS expression cells have already been described 20and support the hypothesis that HCV genotype 1 might induce apoptosis 21,22, this could possibly be the reason for our results since genotype 1 was the most prevalent (73%) in our samples (Table 1). In the other hand, for group 4, we observed a decrease in *FAS* expression in PBMCs, and this was significantly associated with cirrhosis and intense necroinflammation. This result was supported by the hypothesis suggested elsewhere 21that reduced expression of *FAS* receptor is associated with aggressive forms of hepatocellular carcinoma, especially in poorly differentiated tumors that present portal vein or extracapsular invasion, this mechanism could possibly going on in our model. In group 1, we observed a significant increase in *CASP8* expression. Activation of *CASP8* via FAS receptor is an important mechanistic initiator of apoptosis in physiological and pathological conditions, and is extremely important in the pathophysiology of CHC 18.

The activation of caspases can be triggered via death receptors or by mitochondrial dysfunction, with the latter resulting in the release of CYCS 23. The translocation of pro-apoptotic proteins to the mitochondria results in the release of CYCS from outer and inner mitochondrial membrane into the cytosol. In the cytosol, CYCS forms a complex with apoptosis-activating factors, leading to the activation of CASP9, which activates effector caspases.

Group 4 showed a significant increase of CASP9 and CYCS , this found was already supported by at least another group that reported same results associated with significant increases in ALT and AFP levels 24. The indirect markers of fibrosis reflect alterations in hepatic function. A large number of indirect serum markers of fibrosis have been tested over the past few years. They mainly include AST and ALT levels, tested either alone or in combination 25. In our study, we observed significantly increased ALT levels in group 4. The increased ALT levels had been associated to mitochondrial injury, which may be related to HCV infection, and is usually predominant in liver cirrhosis 26,27,28,29. AFP is also important in the diagnosis of hepatocellular carcinoma 30. In the present study, AFP was the most efficient marker for patients in group 4 with liver cirrhosis, confirming the findings of Mohamed *et al*. 31. With regard to the correlation of viral load with biochemical markers, we found a positive and strong correlation of ALT levels in group 4, and a positive and moderate correlation with AFP levels in the same group 32. In groups 2 and 3, we found similar levels of ALT and AFP when compared to control group. Corroborating to these results, *CASP3*, *CASP8*, and *CASP9* mRNA expression levels were also the same as in the control group.

CASP8 and CASP9 are the key initiator caspases activated through the death-receptor and mitochondrial pathways, respectively 33,34, while CASP3 is the critical effector caspase of both pathways. In both groups 1 and 4, we observed a significant increase in CASP3 expression, suggesting a possibly activation of apoptosis.

## Conclusions

In summary, the results of this study corroborate to the body of evidence that HCV could induce the augment of some apoptotic markers suggesting that perhaps apoptosis might be activated through both extrinsic and intrinsic pathways. CASP8 is normally activated through the extrinsic pathway, which is triggered by tumor necrosis factor receptor and FAS receptor 8. In contrast, CASP9 is activated through the intrinsic pathway, which can be initiated by DNA damage and various extracellular stresses 33. Both pathways induce CASP3, causing its cleavage and ultimately apoptosis. This is in agreement with our results showing increased CASP3 levels in both groups 1 and 4, where the levels of CASP8 and CASP9, respectively, were also increased. Nevertheless, further investigations are necessary to evaluated if this caspases are being activated in PBMCs of HCV infected patients.

## Methods

### Patients and histological evaluation of biopsy samples

The present study was prospective and included 50 cases with plasma samples that were positive for RNA-HCV and negative for HIV/HBV by real-time PCR (RT-PCR). Blood samples were collected from patients diagnosed at the Unidade de Referência para Doenças Infecciosas Preveníveis (Urdip) from Faculdade de Medicina do ABC, São Paulo, between June and September 2010. Data included age and gender. We also analyzed liver biopsies (Metavir histologic score) 35, and liver function was estimated by measuring alanine transaminase (ALT) and α-fetoprotein (AFP) levels.

These cases were divided into 4 groups according to the METAVIR scoring system after histological evaluation of 50 paraffin-embedded liver specimens 35. Liver histological staging was based on the degree of fibrosis and inflammatory activity, and the samples were classified into 4 groups: **group 1** (*n* = 13), fibrosis normal and necroinflamation absent; **group 2** (*n* = 14), portal tract fibrosis and mild necroinflamation; **group 3** (*n* = 12), few septa and moderate necroinflammation; and **group 4** (*n* = 11), cirrhosis and intense necroinflammation. We also collected 50 samples of healthy individuals for **control group** (*n* = 50).

### Estimation of AFP and ALT levels

AFP levels were assessed using commercial available ELISA assay kit (IBL America, Minneapolis, MN), according to the manufacturer’s recommendations. The cut-off value of AFP was set at 20 ng/mL, the most commonly used value 36,37.

Plasma ALT values were determined with a Hitachi 7050 automatic analyzer (Hitachi Corp., Tokyo, Japan) using commercial available assay kits (Wako Chemicals). The cut-off value for this assay was also set at 20 mg/mL, as well established in the literature 38.

### Detection of HIV, HCV, and HBV RNA/DNA by magnetic bead isolation and polymerase chain reaction (PCR)

Total RNA/DNA was extracted and purified using magnetic bead technology with the appropriate kit (Chemagic Viral DNA/RNA kit). Three-hundred microliters of plasma was used for each assay, and 100 μL of lysis buffer was added to each sample. The samples were then vortexed and incubated at room temperature for 10 min. Subsequently, 150 μL of the magnetic beads was added, and the mixture was incubated at room temperature for 20 min, with agitation every 10 min. Two-hundred microliters of wash solution was then added, and again, the tubes were vortexed to wash the magnetic beads. Finally, the beads were resuspended with 50 μL of elution buffer. The isolated RNA/DNA (15 μL) was used in PCR reactions for the detection of viral RNA (HIV or HCV) or DNA (HBV). PCR was performed with primers selective for HIV, HCV, and HBV (Table 2) 39. 

**Table 2:** Primer and probe sequences for the studied genes

***Primers and probe***	***Sequences genes***
*Fas R*	*5*^′^- *GGTGCAAGGGTCACAGTGTT*-*3*^′^
*Fas F*	*5*^′^- *TGAAGGACATGGCTTAGAAGTG*-*3*^′^
*Caspase 3R*	*5*^′^- *TGTCGGCATACTGTTTCAGCA*-*3*^′^
*Caspase 3F*	*5*^′^- *GCAGCAAACCTCAGGGAAAC*-*3*^′^
*Caspase 8R*	*5*^′^- *TCGCCTCGAGGACATCGCTCTC*-*3*^′^
*Caspase 8F*	*5*^′^- *CTGCTGGGGATGGCCACTGTG*-*3*^′^
*Caspase 9R*	*5*^′^- *TCTAAGCAGGAGATGAACAAAGG*-*3*^′^
*Caspase 9F*	*5*^′^- *GGACATCCAGCGGGCAGG*-*3*^′^
*Cytochrome CR*	*5*^′^- *TCTCCCCAGATGATGCCTTT*-*3*^′^
Cytochome CF	5^′^- CAAGACTGGGCCAAATCTCC-3
*HCV R*	*5*^′^- *CGCGACCCAACACTACTC*-*3*^′^
*HCV F*	*5*^′^- *CGGGAGAGCCATAGTGGT*-*3*^′^
*HCV PROBE*	*FAM* – *TGCGGAACCGGTGAGTACACC* - *MGB*
*HBV R*	*5*^′^- *GGACAAACGGGCAACATACC*-*3*^′^
*HBV F*:	*5*^′^- *ATGTGTCTGCGGCGTTTTATC*-*3*^′^
*HBV PROBE*:	*FAM* – *TCCTCTTCATCCTGCTGCTATGCCTCATCT* - *MGB*

When we analyzed presence of HIV and HCV, RNA was reverse-transcribed into cDNA by the addition of Superscript III Platinum® One-Step reaction mix containing all necessary reagents for amplification. Amplification was performed using a 7500 Real-Time Sequence Detection System (SDS; ABI Prism 7500; Applied Biosystems, CA). RT-PCR product accumulation was monitored using a TaqMan probe 39,40.

### Standardartization for detection of HCV viral load

Total RNA was extracted and purified using magnetic bead technology with the appropriate kit (Chemagic Viral DNA/RNA kit). The standard used for HCV real-time RT-PCR was the commercial HCV RNA-positive human plasma (Accurun 306; BBI Diagnostics, SeraCare, MA) kit which was tested in triplicate. Ten-fold dilutions of this standard were made down to 310 IU/mL by using normal human plasma, and used in all runs to generate standard curves. Dilutions of this standard were tested with a total of 24 replicates for each point dilution to determine the positive cut-off point or lower limit of detection. RT-PCR product accumulation was monitored using a TaqMan probe 39,40.

### RNA extraction, cDNA synthesis, and quantitative RT-PCR (qRTPCR) of *FAS*, *CASP3*, *CASP8*, *CASP9*, and *CYCS* in PBMCs

Total RNA was purified from PBMCs by phenol and guanidine isothiocyanate-cesium chloride method (Trizol; Life Technologies). RNA was reverse-transcribed into cDNA by addition of a mixture containing 0.5 mg/mL oligo d(T), 10 mM DTT, 0.5 mM dNTPs (Pharmacia Biotech, Sweden), and 200 U of reverse transcriptase (SuperScript RT, Gibco-BRL). Real-time amplification was performed using a 7500 Real-Time Sequence Detection System (SDS; ABI Prism 7500; Applied Biosystems, CA). Reactions were cycled 40 times under the conditions previously determined by conventional PCR. PCR was performed with primers selective for *FAS*, *CASP3*, *CASP8*, *CASP9*, and *CYCS* (Table 2). Results from these experiments, per group, are reported as the relative expression normalized to the *βglobin* housekeeping gene (Applied Biosystems, CA), used as endogenous control 41,42. RT-PCR product accumulation was monitored using a Syber Green probe.

The cycle threshold (Ct) values were subtracted from the housekeeping gene Ct value for each gene to yield ΔCt values. These values were used to carry out statistical comparisons. For graphical representations, the fold-variation was determined using the 2^-(ΔΔCt)^ method, according to published protocols and the manufacturer’s recommendations 43.

### Analysis of CASP8 and CASP9 protein levels using enzyme-linked immune sorbent assay (ELISA)

The plasma levels of CASP8 and CASP9 were assessed using commercially available capture ELISA (IBL America, Minneapolis, MN). Both assays were performed according to the manufacturer’s protocols. The optical density of each sample was determined using an Ultra Microplate Reader (EL808; Bio-Tek Instruments, VT) and expressed as ng/mL 44,45.

### Standardization and analysis of CASP3 protein levels

A microplate was pre-coated with a polyclonal avidin-conjugated antibody specific for CASP3. Standard curve was performed by adding 100uL of CASP3 peptide (0–20 ng/mL) (Abcam Inc, Cambridge, MA) and samples were added to each well. Samples were then incubated for 2 h at room temperature. After this incubation period, samples were aspirated from the well and washed 4 times by filling each well with PBS-Tween 20. Next, 100 μL of antibody with streptavidin conjugated to horseradish peroxidase (Abcam Inc, Cambridge, MA) was added, and the mixture was incubated for 1 h at room temperature before repeating the aspiration/wash procedure. One hundred microliters of tetramethylbenzidine and hydrogen peroxide were then added to each well, and the mixture was incubated for 30 min at room temperature before the addition of 100 μL of 1 N hydrochloric acid to each well. The optical density of each well was determined using a microplate reader set to 450 nm. To determine the CASP3 concentration (ng/mL) of each sample, we first plotted the absorbance value on the y-axis and extended a horizontal line to the standard curve. At the point of intersection, a vertical line was extended to the x-axis, and the corresponding value was the active caspase-3 concentration.

#### HCV genotyping

After HCV RNA extraction using magnetic beads, samples were genotyped through sequencing of 5^′^UTR in a 3500XL genetic analyzer (Applied Biosystems, CA, USA). Primers sequences and protocol were performed as described previously 46.

### Statistical analysis

Data are expressed as the mean ± standard error of the mean (SEM) values. Experimental and control groups were compared using the Student’s t-test for protein levels of CASP8 and CASP9, and ANOVA for the gene expression analysis. The significance level for a null hypothesis was set at 5% (p < 0.05). Pearson’s correlation analysis in Microsoft Excel (Microsoft Corp., WA) was used to determine the relationship between HCV viral load and ALT/AFP levels 47.

The Research Ethics Committee of University Federal of São Paulo/Sao Paulo Hospital examined and approved this research project (no. 0080/10).

## Abbreviations

CHC: Chronic hepatitis C; HCV: Hepatitis C virus; PBMC: Peripheral blood mononuclear cells; HIV: Human immunodeficiency virus; HBV: Hepatitis B virus; RT-PCR: Reverse transcription of polymerase chain reaction; CASP3: Caspase 3; CASP6: Caspase 6; CASP7: Caspase 7; CASP8: Caspase 8; CASP9: Caspase 9; CYCS: Cytochrome c; ALT: Alanine transaminase; AFP: α-fetoprotein.

## Competing interests

The authors declare that they have no conflicts of interest relevant to the manuscript submitted to VIROLOGY JOURNAL.

## Authors’ contributions

GA, CPA, SSA, PRBA, FKR designed and carried out the study. and GA, FRML, MCMC, OHML, PBR, NS, MJCBG and JAB wrote the manuscript. All authors read and approved the final manuscript.
